# Bio-Valorization of Spent Coffee Grounds and Potato Peel as Substrates for *Pleurotus ostreatus* Growth

**DOI:** 10.3390/foods13233774

**Published:** 2024-11-25

**Authors:** Brisa del Mar Torres-Martínez, Rey David Vargas-Sánchez, José Ángel Pérez-Alvarez, Juana Fernández-López, Manuel Viuda-Martos, Martin Esqueda, Javier Germán Rodríguez-Carpena, Félix Joel Ibarra-Arias, Gastón Ramón Torrescano-Urrutia, Armida Sánchez-Escalante

**Affiliations:** 1 Centro de Investigación en Alimentación y Desarrollo (CIAD), Coordinación de Tecnología de Alimentos de Origen Animal (CTAOA), Carretera Gustavo Enrique Astiazarán Rosas 46, Hermosillo 83304, Mexico; brisatorresm@gmail.com (B.d.M.T.-M.); rey.vargas@ciad.mx (R.D.V.-S.); esqueda@ciad.mx (M.E.); gtorrescano@ciad.mx (G.R.T.-U.); 2 Centro de Investigación e Innovación Agroalimentaria y Agroambiental, Universidad Miguel Hernández (CIAGRO-UMH), 03312 Orihuela, Spain; ja.perez@umh.es (J.Á.P.-A.); j.fernandez@umh.es (J.F.-L.); mviuda@umh.es (M.V.-M.); 3 Unidad Académica de Medicina Veterinaria y Zootecnia, Universidad Autónoma de Nayarit, Compostela 67300, Mexico; german.rc@uan.edu.mx; 4 Alta Tecnología Industrial para la Salud Animal, S.A. de C.V. (ATISA), Gabino Barreda 1290, Guadalajara 44430, Mexico; joel.ibarra@atisamx.com

**Keywords:** *Pleurotus*, potato peel, coffee ground, metabolites, antioxidant

## Abstract

*Pleurotus ostreatus*, due to its saprophytic nature, can extract nutrients and bioactive compounds from the substrate on which it is grown. This study aimed to assess the effect of adding spent coffee grounds (SCG) and potato peel (PPW) in the wheat straw substrate formulation to grow over the production indicators, physicochemical, techno-functional, total chemical compounds, and antioxidant properties. Treatments were described as follows: T1, wheat straw at 100%; T2, wheat straw at 80% + 10% of SCG + 10% of PPW; T3, wheat straw at 70% + 15% of SCG + 15% of PPW; T4, wheat straw at 60% + 20% of SCG + 20% of PPW. After *P*. *ostreatus* growth, non-differences were found in production indicators for T1–T4, including biological efficiency, production rate, and yield. With respect to *P*. *ostreatus* dried powders, T1–T4 showed pH values near neutrality concerning soy protein (SP), and the color samples were beige. Also, T2 and T3 exert higher water-holding (WHC) values, while T1–T4 exert higher oil-holding (OHC) and emulsifying capacity (EC) values concerning SP, in dependence on the growth substrate. T1–T4 showed lower swelling (SC) and T1–T3 lower gelling capacity (GC) values. Regarding total chemical compounds and antioxidant properties of *P*. *ostreatus* extracts, growth substrate and solvent extraction have an effect on metabolite content and antiradical and reducing power properties. The multivariate analysis revealed that T2 water extracts exert the highest total tannin (TTC) and protocatechuic acid contents (PAC), as well as the highest antiradical (RCSA) and reducing power (RPA) values. In conclusion, this study demonstrated that using SCG and PPW as a partial substitute for substrate (what straw) enhances the physicochemical, techno-functional, and antioxidant activity of *P*. *ostreatus*.

## 1. Introduction

Bio-valorization of agricultural and industrial waste has emerged as a promising approach for long-term waste management practices and for producing value-added products. This strategy aims to convert waste materials into useful resources, thereby reducing environmental impact while generating economic benefits. Spent coffee grounds (SCG) and potato peel (PPW) are two types of agro-industrial waste that are generated in large quantities [1,2,3]. SCG is a waste product generated during the coffee extraction process in industry. It is characterized by being a nutrient and bioactive compound source, making it suitable for various uses, like compost production and bioenergy, among others [1,2]. In addition, the potato processing industry is an excellent source of bioactive compounds and nutrients that could be used in culinary ingredients, pharmaceuticals, and bioenergy industries, among others [3]. Although often discarded, these materials have a significant potential for bio-valorization. In this context, the cultivation substrates with *Pleurotus ostreatus* (Jacq.) P. Kumm. could be potentiated with these wastes, thus transforming into value compounds.

*Pleurotus ostreatus* (Jacq.) P. Kumm., the scientific name for oyster mushrooms, is an edible mushroom becoming increasingly popular worldwide for consumption and cultivation. Due to its richness in protein, vitamins, minerals, and dietary fiber, this fungal source presents itself as a valuable nutritional option for human consumption [4,5]. It belongs to the genus *Pleurotus* (Fr.) P. Kumm, which comprises several species of saprophytic fungi (i.e., *P*. *eryngii*, *P*. *florida*, *P*. *pulmonarius*, and *P*. *sajor-caju*) that feed on decaying organic matter, especially wood and other lignocellulosic substrates [4].

One biotechnological cultivation method largely used is solid-state fermentation (SSF); with this method, edible mushrooms are grown in a substrate containing a high proportion of solids compared to liquid-state fermentation [5]. The substrate used in the solid medium can vary widely and include materials such as grains, seeds, forestry waste, and agro-industrial waste, among others. During SSF, extracellular enzymes from edible mushrooms are secreted to break down organic matter into minor components that can be absorbed and used as carbon sources and nutrients. This cultivation method has successfully employed agricultural substrates and mushrooms for ligninolytic enzyme production. When applied to fungal species, this fermentation technique enables the synthesis of a diverse range of metabolic products, including various enzymes and bioactive compounds [5,6,7].

Therefore, this research investigated the inclusion of SCG and PPW in the cultivation substrates for *P. ostreatus* to promote the bio-valorization of these underutilized agro-industrial wastes and to determine their effect on productive indicators, physicochemical and techno-functional properties, total chemical compounds, and antioxidant activity of the obtained mushrooms.

## 2. Materials and Methods

### 2.1. Cultivation of the Mushroom

Filamentous *P. ostreatus* strain (IE-8, CIAD-Plant-Based Food Technology Department) was grown on Petri dishes containing potato dextrose medium. The mycelium was incubated at 25 °C for 5 days until it entirely covered the surface (IC403C, Yamato, Tokyo, Japan) and stored at 4 °C. After that, 1/3 of the plate surface with mycelium (approx. 1.5 mg) was incorporated into the inoculum seed contained in plastic bags. Subsequently, approximately 1.5 mg of mycelium was transferred to inoculum seed bags. The seed medium, hydrated overnight, consisted of wheat grains (*Triticum aestivum* L.) that were drained and sterilized (SM300, Yamato, Japan) at 121 °C for 1 h. These inoculated seed bags were kept at 28 °C in darkness until the mycelium thoroughly colonized them, which was evident by a white coloration. Subsequently, wheat straw was used as basal substrate and mixed at different ratios of supplementing residues, as follows: T1, wheat straw at 100%; T2, wheat straw at 80% + 10% of SCG + 10% of PPW; T3, wheat straw at 70% + 15% of SCG + 15% of PPW; T4, wheat straw at 60% + 20% of SCG + 20% of PPW. In this experiment, pre-treated substrates were inoculated with mycelium-coated seed (9:1 ratio). The inoculated substrates were then incubated without light at 28 °C until the fungal growth completely covered the surface. Following complete surface colonization by the fungal growth at 28 °C, the samples were transferred to a cooler, dark environment of 25 °C with a photoperiod of 12 h and a relative humidity between 80 and 90% for 3 weeks for mushroom fructification. Subsequently, production indicators, including biological efficiency, production rate, and yield, were performed [8].

### 2.2. Physicochemical and Techno-Functional Properties

To obtain *P. ostreatus* powder, the fruiting bodies collected from three flushes were dried at 60 °C for 12 h and pulverized at 20 mesh (hammer mill, Pulvex 200, Mexico city, Mexico).

With slight modifications, the AOAC 981.12 procedure was followed to measure the pH values [9]. The dried mushroom powders obtained were homogenized with d-water (1:10 ratio) at 6000 rpm (5 °C) for 1 min, and the values were acquired (pH211, Hanna Instruments Inc., USA). A spectrophotometer (CM508d, Konica Minolta Inc., Tokyo, Japan) was used to quantify the color characteristics of the samples. This instrument measured values for hue (h*), Chroma (C*), red–green color (a*), yellow–blue color (b*), and Lightness (L) under standardized conditions. The setup utilized a D65 illuminant, mimicking daylight, and a 10° observer angle [10].

The mushroom powders were evaluated for their techno-functional properties, including water-holding capacity (WHC), swelling capacity (SC), gelling capacity (GC), oil-holding capacity (OHC), and emulsifying capacity (EC) [11], with slight modifications. Concerning WHC and OHC, samples were homogenized with distilled water or corn oil (1:10 ratio), respectively, at 10,000 rpm for 1 min at 4 °C and kept at 25 °C for 1 h. After, samples were centrifuged at 15,000× *g* for 20 min at 4 °C. The test tubes with the sediments were weighed. The results were expressed as percentages (%). For SC and EC, samples were homogenized with distilled water or corn oil (1:10 ratio) using a graduate test tube, respectively, at 10,000 rpm for 1 min at 4 °C. The samples’ initial and final volume occupied was measured, and the results were expressed as percentages (%). The samples were homogenized with distilled water (1:10 ratio), boiled for 1 h, and cooled at 0 °C for 1 h. After, samples were centrifuged at 15,000× *g* for 20 min at 4 °C. The test tubes with the sediments were weighed. The results were expressed as percentages (%).

### 2.3. Compounds Extraction

The isolation of metabolites from the dried powders was achieved through ultrasound-assisted extraction. This methodology employed a solvent system comprised of water, ethanol, and a 1:10 (*w*/*v*) water–ethanol mixture. The extraction process was conducted under controlled conditions: 40 kHz sonication for 60 min at 25 °C (model 3800, Branson, Dietzenbach, Germany). After extraction, the solution was filtrated through Whatman #1 filter paper utilizing a vacuum pump (model FE-1500, Felisa, Mexico city, Mexico) to remove residual solids. Finally, the filtrate was concentrated via rotary evaporation (RE301BW, Yamato, Japan) at 150 rpm and 65 °C. The concentrated metabolite extracts were then lyophilized (DC401, Yamato, Japan) and stored (−20 °C) in the absence of light [11].

### 2.4. Total Chemical Compounds Content Assays

To determine the total carbohydrate content (TCC) of the extracts, the phenol-sulfuric acid assay was used with slight modifications [12]. A total of 20 μL aliquots of the extracts (10 mg/mL) were combined with 25 μL of 5% (*v*/*v*) aqueous phenolic solution, plus 125 μL of H_2_SO_4_. After incubating at 25 °C with the absence of light, the absorbance of the resulting solution was measured at 490 nm using a Multiskan FC/UV–Vis spectrophotometer (Thermo Scientific, Finland). The TCC was then expressed as milligrams of glucose equivalents per gram of dried extract (mg GE/g).

Like the carbohydrate analysis, the samples’ total protein content (TPC) was assessed using a modified Biuret assay [12]. Briefly, 20 µL aliquots of the samples were combined with 100 µL of Biuret reagent and incubated in the absence of light at 25 °C for 15 min. The absorbance of the resulting solution was then measured at 595 nm using a spectrophotometer. The TPC was subsequently expressed as milligrams of bovine serum albumin equivalents per gram of sample (mg BSA/g).

The extracts’ total phenolic content (TPHC) was quantified using a modified Folin–Ciocalteu assay [13]. Briefly, 20 μL aliquots of the extracts (at a concentration of 10 mg/mL) were mixed with 160 μL of deionized water, 40 μL of Folin–Ciocalteu reagent (2 M), and 60 μL of a 7% (*w*/*v*) sodium carbonate solution. The resulting mixture was incubated in the absence of light at 25 °C for 1 h. Following incubation, the absorbance of the solution was measured at 750 nm using a spectrophotometer. The TPHC was then expressed as milligrams of gallic acid equivalents per gram of dried extract (mg GAE/g).

The total tannin content (TTC) of the extracts was measured employing a modified vanillin assay [14]. A total of 20 μL aliquots of the extracts (at a concentration of 10 mg/mL) were combined with 100 µL of a 1% (*w*/*v*) vanillin solution and 100 µL of 8% (*v*/*v*) hydrochloric acid. The resulting mixture was then incubated in the dark at 25 °C for 20 min. Following incubation, the absorbance of the solution was measured at 500 nm using a spectrophotometer. The TTC was subsequently expressed as milligrams of (+)-catechin equivalents per gram of dried extract (mg CAT/g).

A modified aluminum assay determined the total flavonoid content (TFC) [13]. The procedure combined 20 μL of extract (10 mg/mL concentration) with 130 μL of methanol and 20 μL of 5% (*w*/*v*) aluminum chloride solution. This mixture was then kept in darkness at 25 °C for a 30 min incubation period. Following incubation, the absorbance was measured spectrophotometrically at 415 nm. The results of this analysis were quantified and expressed in terms of mg of quercetin equivalents/g (mg QE/g).

Total chlorogenic acid content (TCGA) was quantified with slight modifications [15]. A total of 20 μL of extract (10 mg/mL concentration) were combined with 100 μL of 0.17 M urea, 100 μL of 0.1 M glacial acetic acid, and 250 μL of distilled water. This mixture was then homogenized with equal volumes (250 μL each) of 0.14 M sodium nitrite and 1 M sodium hydroxide. The resulting solution underwent centrifugation (Sorvall ST18R, Thermo Fisher Scientific, Waltham, MA, USA) at 2250 ×g for 10 min at 4 °C. Subsequently, the absorbance of the supernatant was measured at 510 nm. Results were expressed as mg chlorogenic acid equivalents/g (mg CGA/g).

### 2.5. HPLC Assay

An HPLC system (model 1100 series, HP, Waldborn, Germany) with a diode-array detector (DAD G-1315A) equipped with a reversed-phase column (300 × 78 mm, 5 mm; at 30 °C) was used for the analysis of phenolic compounds. Formic acid (5%, *v*/*v*) and methanol were used as the mobile phase; the sample injection volume was set to 10 μL at a flow rate of 1.0 mL/min, and the absorbance was measured at 340 nm and 280 nm. All compounds were identified by the retention times of each standard and quantified by their peak areas related to those of standard curves [16].

### 2.6. Antioxidant Assays

With slight modifications, the free-radical scavenging activity (FRSA) was measured by the DPPH assay [17]. The extracts (20 μL, 10 mg/mL) were mixed with 100 μL DPPH ethanol solution (300 μM; 1,1-diphenyl-2-picrylhydrazyl). The resultant mixture was incubated at 25 °C for 30 min in the absence of light, and the absorbance was measured at 517 nm. Butylhydroxytoluene (100 μg/mL; BHT) was used as a positive control. Results were expressed as inhibition percentage: [(DPPH absorbance at 0 min) − (DPPH absorbance + each extract at 30 min)/(DPPH absorbance at 0 min)] × 100.

With slight modifications, the ABTS assay determined the radical cation scavenging activity (RCSA) [17]. The extracts (20 μL, 10 mg/mL) were mixed with 180 μL of ABTS solution (2,2-Azino-bis (3-ethylbenzothiazoline-6-sulfonic acid) diammonium salt). The resultant mixture was incubated at 25 °C for 8 min in the absence of light, and the absorbance was measured at 730 nm. BHT (100 μg/mL) was used as a positive control. Results were expressed as inhibition percentage: [(ABTS absorbance at 0 min) − (ABTS absorbance + each extract at 30 min)/(ABS absorbance at 0 min)] × 100.

With slight modifications, the ferric-reducing antioxidant power (FRAP) was determined [18]. The extracts (20 μL, 10 mg/mL) were mixed with 180 μL of FRAP solution [10:1:1, 300 mM buffer sodium acetate in glacial acetic acid at pH 3.6 and 2,4,6-tripyridyl-s-triazine (10 mM) in hydrochloric acid (40 nM) and iron chloride (20 mM)]. The reaction mixture was incubated at 25 °C for 8 min in the absence of light, and the absorbance was measured at 595 nm. BHT (100 μg/mL) was used as a positive control. Results were expressed as mg of Fe^2+^ equivalent/g (mg Fe^2+^/g).

With slight modifications, the reducing power ability (RPA) was determined by the ferricyanide/Prussian blue assay [18]. The extracts (20 μL, 10 mg/mL) were mixed with 300 μL of phosphate buffer (0.2 M, pH 6.6) and 500 μL of potassium ferrocyanide (1%, *w*/*v*) and incubated at 50 °C for 20 min with the absence of light. The obtained solution was homogenized with 500 μL of trichloroacetic acid (10%, *w*/*v*) and centrifuged at 2300 ×g at 4 °C for 10 min. Subsequently, the supernatant (100 μL) was homogenized with 100 μL of iron chloride (0.1%, *w*/*v*), and the absorbance was measured at 700 nm. BHT (100 μg/mL) was used as a positive control. Results were expressed as absorbance at 700 nm.

### 2.7. Statistical Analysis

The data were presented as the mean ± standard deviation (SD). The data collected on production metrics, physicochemical characteristics, and techno-functional attributes of the cultivated mushrooms underwent statistical evaluation using one-way analysis of variance (ANOVA). In contrast, metabolite content and antioxidant activity results were subjected to a two-way ANOVA, considering the treatments and solvent extractions as fixed effects and their interaction. The Tukey test with a significance level of *p* < 0.05 was conducted. A principal component analysis (PCA) was performed to evaluate the relationships among variables used in the SPSS software (version 21).

## 3. Results and Discussion

### 3.1. Production Indicators, Physicochemical and Techno-Functional Values

Production indicators and physicochemical and techno-functional properties of *P. ostreatus* cultivated in different agro-industrial wastes are presented in Table 1.

The data showed that increasing the amount of residue in the substrate led to significant improvements in biological efficiency, production rate, and yield (*p* < 0.05). Furthermore, the analysis indicated that samples from T1 showed higher pH values than SP (*p* < 0.05). For color parameters, results indicate that the lowest L* values were observed in SP and T3 samples. At the same time, T4 exhibited the minimum values for a*, b*, and C* while the maximum for h* (*p* < 0.05). According to the obtained RGB values and HEX code, the assigned color names were Dark Beige (T1, T2, and T3), Bone (T4), and Lion (SP).

Likewise, values greater than 40% of biological efficiency have been reported after cultivating *P*. *ostreatus* IE-8 on mixtures of viticulture residues [8]. Another study reported that after the growth of *P*. *ostreatus* ECS-1123 and *Pleurotus djamor* (Rumph. ex Fr.) Boedijn (ECS-123) in agro-industrial residues (pangola grass, coffee pulp, and corn cob), the biological efficiency values ranged between approx. 30 and 63%, while in contrast, the production rate and yield values ranged between 0.1–0.6% and 6.5–18.8%, respectively [19]. Also, an increase in biological efficiency was reported in *P*. *djamor* (IE-218 and IE-121), *P*. *ostreatus* (IE-49 and IE-38), and *Pleurotus pulmonarius* (Fr.) Quél. (IE-25 and IE-137) grown in substrates containing coffee pulp, respecting the substrates with wheat straw (substrate control) [20]. Biological efficiency values greater than 40% were reported in the cultivation of *Pleurotus eryngii* (DC.) Quél., *P. ostreatus*, *and Pleurotus sajor-caju* (Fr.) Fr. *on agro-industrial substrates* (banana, mango, melon rind, orange, peels of avocado, pineapple, and wheat straw) [21].

In addition, in the food industry, the pH and temperature are parameters that can affect the texture and color of edible mushrooms and, consequently, consumer acceptance. Hence, it is recommended to avoid acidification of the material and drying to reduce the microstructure degradation, extend its shelf life, and prevent nutrient loss [22,23]. In agreement, the powders from *P*. *ostreatus* and *P*. *pulmonarius* grown in wheat straw showed pH values near neutrality; however, the type of color presented was Sorrell Brown and Tan, respectively [11]. A previous study found that *Pleurotus* sp. mushrooms grown on various agro-industrial residues exhibited a powder with a pH greater than 6.0 (bean pods, coffee husk, corn straw, wheat bran, wood chips, wood sawdust, and rice husks; unknown proportion), while the reported color for these samples was Tan [24].

**Figure 1:**
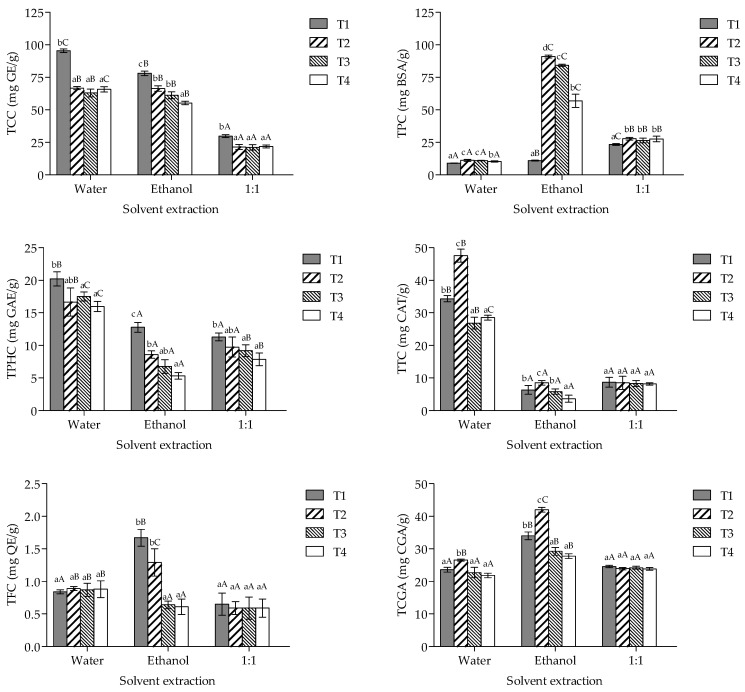
Total chemical compounds content of the extracts from *P*. *ostreatus* cultivated on spent coffee grounds and potato peel residues. TCC, total carbohydrate content; TPC, total protein content; TPHC, total phenolic content; TTC, total tannin content; TFC, total flavonoid content; TCGA, total chlorogenic acid content. T1, wheat straw at 100%; T2, wheat straw at 80% + 10% of SCG + 10% of PPW; T3, wheat straw at 70% + 15% of SCG + 15% of PPW; T4, wheat straw at 60% + 20% of SCG + 20% of PPW. Lowercase and capital letters stipulate differences between treatments × solvent extraction (*p* < 0.05).

Additionally, Figure 1 shows the techno-functional properties of *P*. *ostreatus* cultivated in different agro-industrial residues. Analysis of the results indicated that samples from T2 and T3 exhibited the highest WHC values, while T1–T4 exerted higher OHC and EC values than SP (*p* < 0.05). In addition, SP exhibited the highest SC values, followed by T3 and T4. In terms of GC, both T4 and SP displayed the highest values (*p* < 0.05).

Powders obtained from natural sources are characterized by having food applications as techno-functional ingredients to formulate and stabilize their structures, for example, solubility properties, emulsification, foaming, etc.; however, the techno-functional properties of these ingredients depend on the type, quantity, chemical composition, and extrinsic factors during processing [25]. In agreement, the powders from *P*. *ostreatus* and *P*. *pulmonarius* grown in wheat straw showed higher OHC and lower GC than SP; in contrast, lower WHC than SP was recorded, while no differences were demonstrated in SC [11]. Another study reports that the powder of *Pleurotus* sp. cultivated in agro-industrial residues exerts water and fat retention, as well as swelling and emulsifying properties [24]. Also, a previous study reported an increase in WHC of the powder from *P*. *ostreatus* AMRL144 and AMRL150 grown in substrates containing barley and oat straw concerning a protein concentrate; however, no differences were found in OHC and EC values concerning the control [26].

### 3.2. Total Chemical Compounds Content Values

The metabolite content of the extracts obtained from *P. ostreatus* cultivated in different agro-industrial residues is illustrated in Figure 1. The results showed a statistically significant interaction between the treatment and the solvent extraction (*p* < 0.001) on evaluated parameters. Concerning primary metabolites, water extracts (T1 > T2–T4) showed the highest TCC values, while ethanol extracts (T2 > T3 > T4) demonstrated the highest TPC values among all the extracts analyzed (*p* < 0.05). Regarding polyphenols, water extracts showed higher TPHC (T1 > T2–T4) and TTC values (T2 > T1 > T3 and T4), while ethanol extracts from T1 and T2 contained the maximum levels of TFC. In addition, ethanol extracts (T2 > T1 > T3 and T4) showed higher TCGA values.

**Figure 2:**
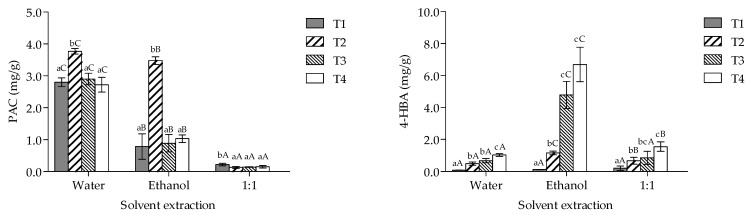
HPLC-polyphenol quantification of the extracts from *P*. *ostreatus* cultivated on an added substrate with spent coffee grounds and potato peel residues. PAC, protocatechuic acid; 4-HBA, 4-hydroxybenzoic acid. T1, wheat straw at 100%; T2, wheat straw at 80% + 10% of SCG + 10% of PPW; T3, wheat straw at 70% + 15% of SCG + 15% of PPW; T4, wheat straw at 60% + 20% of SCG + 20% of PPW. Lowercase and capital letters stipulate differences between treatments × solvent extraction (*p* < 0.05).

The quantitative analysis of polyphenols, performed via HPLC, for extracts derived from *P. ostreatus* growth in different agro-industrial residues is illustrated in Figure 2. The results demonstrated a statistically significant effect of the treatment and solvent extraction (*p* < 0.001) on the individual polyphenol content. According to the results, water extracts (T2 > T1, T3, and T4) showed the highest PAC values, while ethanol extracts showed the highest 4-HBA (T3 and T4 > T2 > T1) values (*p* < 0.05).

*Pleurotus* species have been considered an essential source of primary and secondary metabolites. Regarding primary metabolites, carbohydrates are involved in structural body components, except for free sugars, which are necessary for energy metabolism. Non-protein nitrogen compounds present in mushrooms are derived from sources such as chitin, nucleic acids, and amino acids. Concerning secondary metabolites, phenolic compounds have been extensively investigated in this species [4,27]. In agreement, an earlier study reported a decline in TCC values and an increase in TPC values of *P*. *ostreatus* grown in corn cob substrates containing herb residues [28]. Also, it was reported an increase in the TPC values of two *P*. *ostreatus* strains (AMRL144 and AMRL150) grown in substrates containing barley and oat straw respect substrates with wheat straw (substrate control) [26].

The presence of phenolic compounds has been reported in *Pleurotus* spp. cultivated in agro-industrial residues [24]. In agreement, a previous study reported an increase in TPHC values of the water extract from *P*. *ostreatus* cultivated in corn cob substrates containing herb residues [28]. A higher TPHC value was reported in the water extract from *P*. *ostreatus* grown in substrates containing avocado, mango, oranges, and pineapple peels compared to the extract from the mushroom growing in wheat straw (substrate control); also, the water extract from *P*. *sajor-caju* grown in substrates with avocado, banana, and mango peels showed the highest TPHC values. Meanwhile, the water extract from *P*. *eryngii* grown in substrates containing mango, orange, and pineapple peels showed an increase in TPHC values. In contrast, it was demonstrated that TPHC values were reduced in the water extract from *P*. *eryngii* cultivated in the substrate with avocado peels [21]. A previous study reported higher TPHC and TFC values in the water than methanol extract from *P. ostreatus* grown on a range of ligneous media, encompassing beech, poplar, walnut, linden, oak, and aspen [29]. Also, there was an increase in TPHC, TFC, and TTC values of the water extract from *P*. *pulmonarius* grown in substrates containing palm bunches and corn cobs [30]. Another study found that *P*. *djamor* grown in substrates containing a mixture of banana leaves and sugarcane bagasse had an increase in TPHC and TFC values in the water–ethanol extract compared to those grown on just banana leaves; however, no differences were shown in TCC and TPC values [31]. Regarding solvent extraction, higher TPHC values have been reported in water than in ethanol extract from *P*. *ostreatus* [32]. Conversely, a previous study reported a decrease in TPHC and TTC values of the methanol extract from *P. ostreatus* grown in substrates containing a mixture of wood sawdust (*Castanea sativa* and *Populus orientalis*) respect substrates with only *C*. *sativa* as substrate [33]. Regarding individual phenolic compounds, P. ostreatus strains have identified some phenolic acids, including hydroxybenzoic, protocatechuic, syringic, vanillic, caffeic, cinnamic, and ferulic [34].

### 3.3. Antioxidant Activity Values

**Figure 3:**
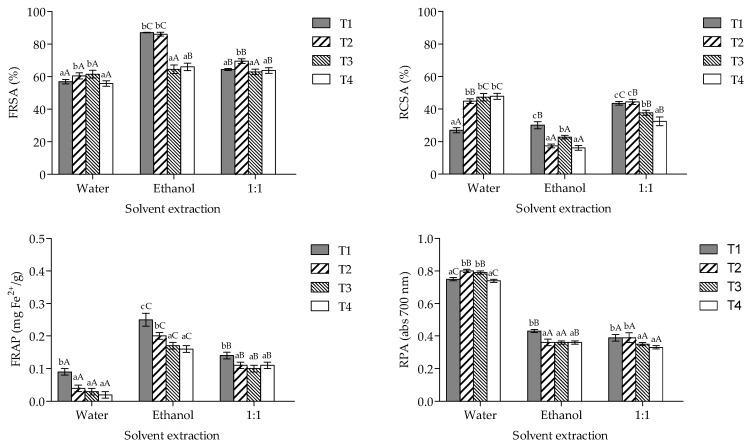
Antioxidant activity of the extracts from *P*. *ostreatus* cultivated on spent coffee grounds and potato peel residues. FRSA, free-radical scavenging activity; RCSA, radical cation scavenging activity; FRAP, ferric-reducing antioxidant power; RPA, reducing power ability. T1, wheat straw at 100%; T2, wheat straw at 80% + 10% of SCG + 10% of PPW; T3, wheat straw at 70% + 15% of SCG + 15% of PPW; T4, wheat straw at 60% + 20% of SCG + 20% of PPW. Lowercase and capital letters stipulate differences between treatments × solvent extraction (*p* < 0.05).

Figure 3 shows the antioxidant activity of the extracts obtained from *P*. *ostreatus* cultivated in different agro-industrial residues. Analysis revealed a statistically significant effect of the interaction between the treatments and the solvents used for extraction (*p* < 0.001) on evaluated parameters. According to the obtained results, ethanol extracts showed higher FRSA (T1 = T2 > T3 and T4) and FRAP values (T1 > T2 > T3 and T4), while water extracts showed higher RCSA (T2–T4 > T1) and RPA (T2 and T3 > T1 and T4) values (*p* < 0.05).

In agreement, an earlier study reported an increase in the FRSA and FRAP values of the methanol extract from *P. ostreatus* grown in substrates containing a mixture of wood sawdust (*Castanea sativa* and *Populus orientalis*) respect substrates with only *C*. *sativa* as substrate [33]. A previous study found that *P. ostreatus* mushrooms grown on corn cobs with added herb residues exhibited an increase in the FRSA of their water extracts [28]. Higher FRSA values were reported in the water extract of *P*. *eryngii*, *P*. *sajor-caju*, and *P*. *ostreatus* cultivated in substrates added with mango, orange, avocado, and pineapple peels than the extract obtained from the control substrate. Furthermore, previous research has shown that the water extract of *P*. *ostreatus* grown in the substrate with melon rind exerted the highest FRSA [21]. It was observed a decrease in the RPA values of the methanol extract from *P*. *ostreatus* grown in substrates containing a mixture of sugarcane bagasse and wheat bran with respect to substrates with sugarcane bagasse [35]. The analysis indicated that the water–methanol extract of *P*. *ostreatus* grown in coconut coir and pine sawdust exerts higher FRSA and RCSA values than the mushroom grown in wastepaper, while *P*. *ostreatus* grown in coconut coir showed the highest FRAP values [36]. Also, there was an increase in FRSA and RCSA values of the water extract from *P*. *pulmonarius* grown in substrates containing palm bunches and corn cobs [30]. Another study reported an increase in RCSA values in the water–ethanol extract from *P*. *djamor* grown in substrates containing a mixture of banana leaves and sugarcane bagasse to respect the substrates with only banana leaves; however, no differences were shown in FRSA values [31]. Regarding solvent extraction, higher FRSA and FRAP values have been reported in ethanol than in water extract from *P*. *ostreatus* [32].

### 3.4. Multivariate Analysis of Metabolites vs. Antioxidant Values

**Figure 4:**
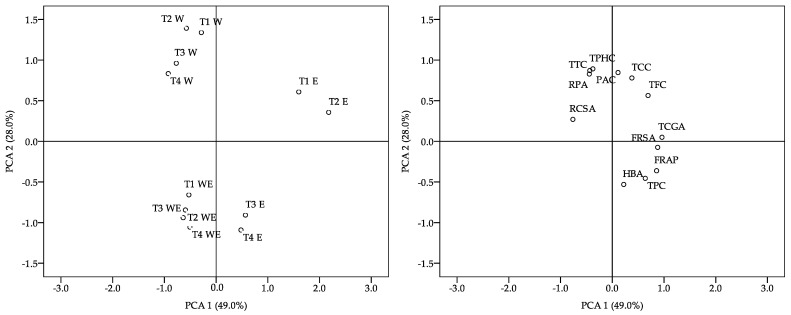
PCA of treatment and analyzed parameters. TCC, total carbohydrate content; TPC, total protein content; TPHC, total phenolic content; TTC, total tannin content; TFC, total flavonoid content; TCGA, total chlorogenic acid content; PAC, protocatechuic acid; 4-HBA, 4-hydroxybenzoic acid; FRSA, free-radical scavenging activity; RCSA, radical cation scavenging activity; FRAP, ferric-reducing antioxidant power; RPA, reducing power ability. T1, wheat straw at 100%; T2, wheat straw at 80% + 10% of SCG + 10% of PPW; T3, wheat straw at 70% + 15% of SCG + 15% of PPW; T4, wheat straw at 60% + 20% of SCG + 20% of PPW. W, water; E, ethanol; WE, water–ethanol.

Figure 4 shows the PCA of treatments and analyzed parameters. The first two principal components explained a significant portion (77.0%) of the total variation, with contributions of 49.0% and 28.0% from the first and second components, respectively. The PCA demonstrated that T2 W samples, grouped in the graph towards the upper left quadrant, presented the highest TTC, PAC, RPA, and RCSA values.

## 4. Conclusions

After *P*. *ostreatus* growth, non-differences were found in production indicators for T1–T4, including biological efficiency, production rate, and yield. Concerning *P*. *ostreatus* dried powders, T1–T4 showed pH values near neutrality concerning soy protein (SP), and the color samples were beige. Also, T2 and T3 exert higher water-holding (WHC) values, while T1–T4 exert higher oil-holding (OHC) and emulsifying capacity (EC) values concerning SP, in dependence on the growth substrate. T1–T4 showed lower swelling (SC) and T1–T3 lower gelling capacity (GC) values.

Regarding the chemical composition and antioxidant properties of *P*. *ostreatus* extracts, growth substrate and solvent extraction affect metabolite content and antiradical and reducing power properties. The multivariate analysis revealed that T2 water extracts exert the highest total tannin (TTC) and protocatechuic acid contents (PAC), as well as the highest antiradical (RCSA) and reducing power (RPA) values.

In conclusion, this study demonstrated that using SCG and PPW as a partial substitute for substrate (what straw) enhances the physicochemical, techno-functional, and antioxidant activity of *P*. *ostreatus*. Therefore, cultivating *P. ostreatus* in solid media using partially agro-industrial residues as substrate may be a promising strategy for obtaining new food additives.

## Figures and Tables

**Table 1:** Production indicators and physicochemical and techno-functional properties of *P*. *ostreatus* cultivated on spent coffee grounds and potato peel residues.

Item	Treatments				
	T1	T2	T3	T4	SP
*Production indicators*					
Biological efficiency	45.92 ± 12.93 ^a^	49.52 ± 8.51 ^a^	57.38 ± 14.04 ^a^	58.29 ± 11.00 ^a^	-
Production rate	0.98 ± 0.28 ^a^	1.05 ± 0.18 ^a^	1.22 ± 0.30 ^a^	1.24 ± 0.23 ^a^	-
Yield (%)	1.58 ± 0.66 ^a^	1.90 ± 0.50 ^a^	1.73 ± 0.87 ^a^	2.20 ± 0.52 ^a^	-
*Physicochemical properties*					
pH	6.53 ± 0.03 ^b^	6.44 ± 0.06 ^ab^	6.50 ± 0.05 ^ab^	6.50 ± 0.05 ^ab^	6.45 ± 0.02 ^a^
L*	82.62 ± 0.76 ^b^	82.88 ± 0.47 ^b^	80.39 ± 0.60 ^a^	83.41 ± 0.57 ^b^	80.15 ± 0.28 ^a^
a*	0.30 ± 0.15 ^b^	0.16 ± 0.09 ^b^	0.37 ± 0.18 ^c^	−0.08 ± 0.06 ^a^	2.58 ± 0.17 ^d^
b*	12.01 ± 0.21 ^b^	14.03 ± 0.20 ^c^	12.36 ± 0.22 ^b^	11.28 ± 0.22 ^a^	20.29 ± 0.46 ^d^
C*	12.02 ± 0.21 ^b^	14.03 ± 0.20 ^c^	12.36 ± 0.22 ^b^	11.28 ± 0.22 ^a^	20.45 ± 0.48 ^d^
h*	88.58 ± 0.70 ^b^	89.35 ± 0.36 ^b^	88.27 ± 0.82 ^b^	90.39 ± 0.30 ^c^	82.75 ± 0.30 ^a^
RGB	(213, 205, 183)	(215, 206, 180)	(207, 199, 176)	(214, 207, 187)	(215, 196, 161)
HEX code	#d5cdb7	#d7ceb4	#cfc7b0	#d6cfbb	#d7c4a1
Color name	Dark Beige	Dark Beige	Dark Beige	Bone	Lion
*Techno-functional properties*					
WHC	74.21 ± 0.58 ^b^	77.46 ± 0.54 ^d^	78.99 ± 1.00 ^d^	75.78 ± 0.81 ^c^	71.02 ± 0.42 ^a^
OHC	71.14 ± 1.52 ^b^	71.80 ± 1.99 ^b^	73.58 ± 0.42 ^b^	72.20 ± 0.64 ^b^	50.75 ± 2.51 ^a^
SC	72.73 ± 1.00 ^a^	69.44 ± 4.81 ^a^	78.41 ± 3.94 ^b^	77.27 ± 2.50 ^b^	90.00 ± 1.01 ^c^
EC	60.00 ± 1.00 ^b^	60.00 ± 1.01 ^b^	59.99 ± 1.00 ^b^	60.01 ± 0.99 ^b^	1.00 ± 0.11 ^a^
GC	N.D.	N.D.	N.D.	60.01 ± 0.96 ^a^	60.00 ± 1.06 ^a^

Results are described as mean ± SD. RGB, red–green–blue; WHC, water-holding capacity; OHC, oil-holding capacity; SC, swelling capacity; EC, emulsifying capacity; GC, gelling capacity. N.D., not detectable. T1, wheat straw at 100%; T2, wheat straw at 80% + 10% of SCG + 10% of PPW; T3, wheat straw at 70% + 15% of SCG + 15% of PPW; T4, wheat straw at 60% + 20% of SCG + 20% of PPW; SP, soy protein. In the same row, treatment differences are indicated by lowercase (*p* < 0.05).

## Data Availability

The original contributions presented in this study are included in the article. Further inquiries can be directed to the corresponding author.
